# Preoperative predictors of dysphagia after transoral surgery

**DOI:** 10.1007/s10147-021-01860-9

**Published:** 2021-01-18

**Authors:** Kazunori Fujiwara, Kenkichiro Taira, Ryohei Donishi, Satoshi Koyama, Tsuyoshi Morisaki, Takahiro Fukuhara, Hiromi Takeuchi

**Affiliations:** Division of Otolaryngology, Head and Neck Surgery, Department of Sensory and Motor Organs, School of Medicine, Tottori University Faculty of Medicine, Yonago, 683-8504 Japan

**Keywords:** Transoral surgery, Dysphagia, Videofluoroscopy, Swallowing function, Predictive factor, Aspiration

## Abstract

**Background:**

Transoral surgery (TOS) has been used to remove pharyngeal and laryngeal cancers with the objective of improving functional without worsening survival. However, there is a risk of postoperative dysphagia, which can severely impair quality of life. The aim of this study was to evaluate the preoperative predictive factors for postoperative dysphagia in patients undergoing TOS.

**Methods:**

One hundred and twenty patients who underwent TOS were evaluated in this study. The degree of dysphagia was evaluated using the Functional Outcome Swallowing Scale (FOSS) both preoperatively and 3 months postoperatively. Those whose FOSS stage was maintained postoperatively were classified into the FOSS-M group, while those with increased FOSS stage postopratively were classified into the FOSS-I group. The following parameters were assessed before surgery: age, weight, height, body mass index (BMI), forced expiratory volume in 1 s, and history of head and neck radiotherapy. Videofluoroscopy (VF) was performed preoperatively to evaluate swallowing function using the Penetration-Aspiration Scale (PAS).

**Results:**

The BMI of the FOSS-M group was significantly higher than that of the FOSS-I group. A history of radiotherapy was significantly more common in the FOSS-I group than in the FOSS-M group. Finally, preoperative PAS in the FOSS-M group was lower than that in the FOSS-I group.

**Conclusion:**

This study suggested that patients with preoperative aspiration detected using VF might develop postoperative dysphagia severely. In addition, preoperative low BMI and a history of previous radiotherapy for head and neck cancer were associated with postoperative dysphagia. Objective examinations such as VF should be performed preoperatively.

## Introduction

Head and neck squamous cell carcinoma (HNSCC) is treated with surgery, radiotherapy, or chemotherapy, or a combination thereof, depending on location and stage. Recently, concurrent chemotherapy was administered as a primary modality in cases of early-stage pharyngeal cancer. Meta-analyses have demonstrated that altered fractionation regimens and/or the addition of chemotherapy not only improve survival, but also result in a significant increase in treatment-related toxicities, particularly acute mucositis, xerostomia, and long-term dysphagia [1–3]. The rate of gastrostomy tube dependence in patients treated with chemoradiotherapy has been reported to range from 9 to 39% [4].

Minimally invasive surgery techniques for HNSCC continue to be frequently reported in the head and neck literature, driven by the desire to offer a less morbid alternative to chemoradiation. These techniques include transoral laser microsurgery and, more recently, transoral robotic surgery (TORS). TORS was first introduced by Weinstein et al. in 2005 in their case report of supraglottic laryngectomy performed in a canine model [5]. TORS with the da Vinci Surgical System has been used to remove pharyngeal and laryngeal cancers with the objective of improving functional and aesthetic outcomes without worsening survival [6–8]. Additional transoral surgery (TOS) techniques have been developed, such as transoral videolaryngoscopic surgery (TOVS) and endoscopic laryngopharyngeal surgery (ELPS). One of the advantages of TOS over chemoradiotherapy is that swallowing function tends to be preserved. TOS was shown to result in a lower rate of percutaneous endoscopic gastrostomy (PEG) dependence and a higher rate of oral intake compared to chemoradiotherapy [9].

While dysphagia is not common following TOS, it can severely impair quality of life. There have been few reports about the risk of dysphagia associated with TOS. The aim of this study was to evaluate preoperative predictive factors for postoperative dysphagia in patients undergoing TOS.

## Methods

### Patients

Between April 2010 and March 2019, 160 patients who presented with benign or malignant lesions of the oropharynx, supraglottic larynx, or hypopharynx underwent TOS at our hospital. Of these 160 patients, 120 were evaluated in this study. Table 1 shows the distribution of indications for TOS.

**Table 1:** Types of cancer in patients who underwent transoral surgery

Disease	Number
Hypophaeyngeal cancer	64
Oropharyngeal cancer	30
p16+	5
p16−	25
Supraglottic cancer	6
Cervical esophageal cancer	2
Other	18

Preoperatively, all patients underwent the following: endoscopic examination; computed tomography of the throat, neck, and chest, with contrast; ultrasonography; and videofluoroscopy. The inclusion criteria of this study were as follows: (1) age 20 years or older; (2) ECOG performance status of 0–2; and (3) diagnosis of oropharyngeal, hypopharyngeal, supraglottic, or cervical esophageal carcinoma classified preoperatively as Tis, T1, or T2, and N0, N1, N2a, N2b, or N2c, according to the 8th UICC staging system. In cases of oropharyngeal squamous cell carcinoma, the presence of p16 was evaluated immunohistochemically and patients were classified as p16 positive and p16 negative/unknown. The exclusive criteria of TOS were as follows: anticipated resection more than halfway around the pharynx, visible invasion into the laryngeal cavity, and no oral intake preoperatively. Patients who had a tracheostoma preoperatively and those who refused surgery were excluded.

### Evaluated parameters

A number of parameters were evaluated preoperatively, including age, weight, height, and body mass index (BMI). Respiratory function parameters such as forced expiratory volume in 1 s (%FEV1), expressed as a percentage of the flow-volume curve, were evaluated using a spirometer. We also determined whether each patient had a history of neck or adjuvant radiotherapy. Degree of dysphagia was evaluated by the Functional Outcome Swallowing Scale (FOSS) both preoperatively and 3 months postoperatively. The FOSS categorizes swallowing function into 6 stages, as follows: stage 0, normal function and asymptomatic; stage 1, normal function with episodic or daily symptoms of dysphagia; stage 2, compensated abnormal function manifested by significant dietary modifications or prolonged mealtimes but without weight loss or aspiration; stage 3, decompensated abnormal function with weight loss of < 10% of body weight over 6 months caused by dysphagia, or daily cough, gagging, or aspiration during meals; stage 4, severely decompensated abnormal function with weight loss of > 10% of body weight over 6 months caused by dysphagia, or severe aspiration with bronchopulmonary complications; and stage 5, nonoral feeding for all nutrition. Patients were divided into two groups according to the presence of impaired postoperative swallowing function. Those whose swallowing function and FOSS stage were maintained postoperatively were classified into the FOSS-M group, while those with worsened swallowing function postoperatively, and thus increased FOSS stage, were classified into the FOSS-I group.

Videofluoroscopy was performed preoperatively to evaluate swallowing function, which was graded using the Penetration-Aspiration scale (PAS) by two ENT doctors and two speech therapists. The PAS is scored as follows: score 1, material does not enter the larynx or trachea; score 2, material enters the larynx and is cleared; score 3, material enters the larynx and is not cleared; score 4, material contacts the true vocal folds and is cleared; score 5, material contacts the true vocal folds and is not cleared; score 6, material enters the trachea and is spontaneously cleared into the larynx or pharynx; score 7, material enters the trachea and is not cleared despite effort; score 8, material enters the trachea and there is no attempt to clear.

### Surgical procedure

This study included three TOS procedures, specifically TOVS, ELPS, and TORS. TOVS was performed according to the procedure reported by Tomifuji et al. An FK-WO TORS Laryngo-Pharyngoscope Retractor (Olympus; Tokyo, Japan) was positioned to provide sufficient working space. A flexible endoscope capable of angulation in four directions (Visera LTF-type VP, Olympus) was inserted through the oral cavity while an assistant held and manipulated the endoscope to achieve visualization of the surgical field. The operator employed a single-use electrosurgical knife with radiofrequency alternating current (KD-600, Olympus) to resect the tumor, and used forceps designed for endoscopic surgery (AdTec monopolar; AESCULAP) to grasp the tissue. If hypopharyngeal exposure was inadequate with the FK-WO retractor, ELPS was performed using a curved laryngoscope and forceps; the use of this instrumentation was the only difference between ELPS and TOVS. The selection between TOVS and TORS was based on patient preference.

TORS was performed according to a previously reported procedure. A FK-WO TORS laryngo-pharyngoscope retractor was positioned to provide an adequate surgical field. A 3D endoscope was inserted through the oral cavity and two articulated robotic instruments were inserted on each side of the endoscope. A 0° 3D endoscope was selected for the soft palate and lateral wall of the oropharynx, and a 30° 3D endoscope was utilized for the tongue base and hypopharynx. For instrumentation, a monopolar spatula was used on the affected side, and Maryland forceps were used on the intact side. The 5-mm tumor margin was determined with the aid of a narrow-band image, and the resection was performed en bloc. After the surgery, a nasogastric tube was inserted. For 2 days, patients were tube fed and ingested nothing by mouth.

All patients provided informed consent. This study was approved by the institutional review board of our hospital (IRB number 2723).

### Statistical analysis

All values are presented as means ± SE. We used Prism 8 for Mac to analyze the data. The Mann–Whitney *U* test was used to evaluate differences in age and BMI, as well as differences in parameters such as preoperative PAS and FEV between the FOSS-M and FOSS-I groups. For statistical analysis of the history of radiotherapy and postoperative radiotherapy, the Fisher exact test was used.

## Results

### Patients

**Fig. 1:**
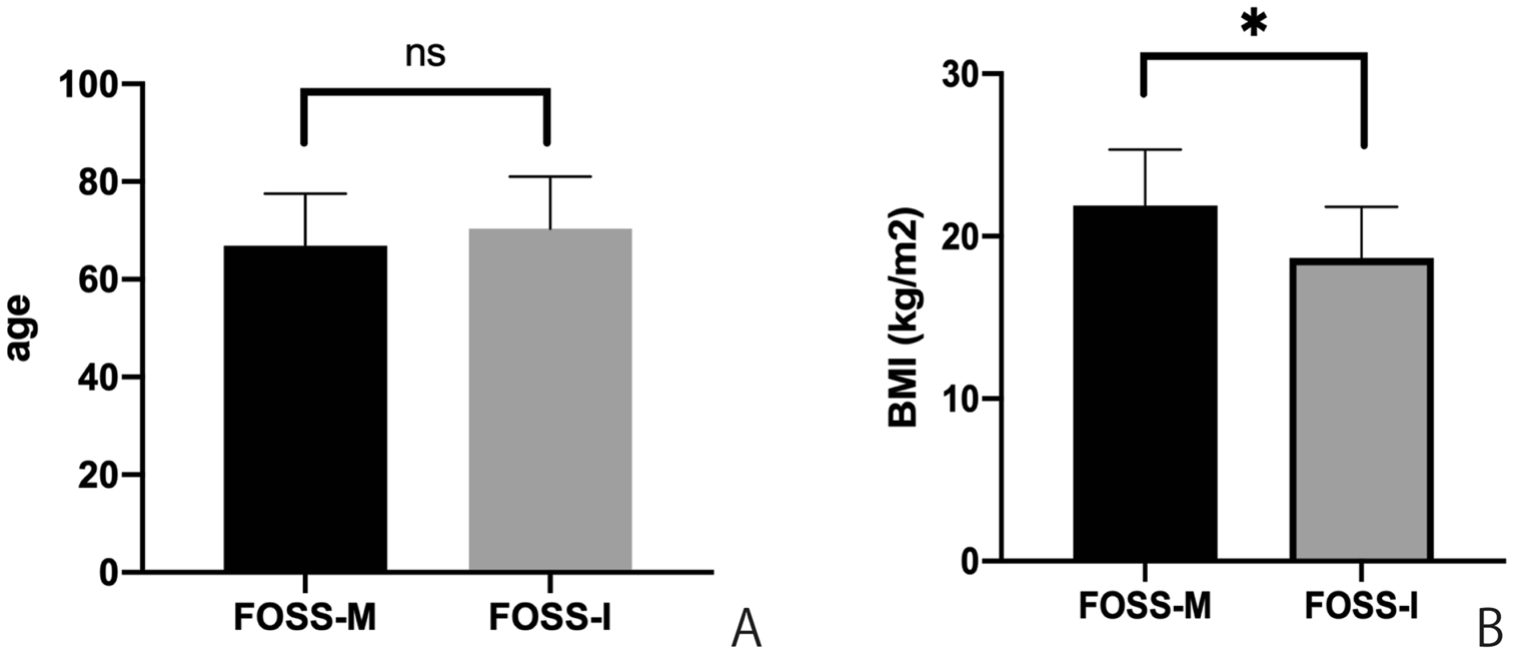
Comparison of age and body mass index (BMI) between the two groups. The mean age of the FOSS-M group was 66.9 ± 1.0 years and that of the FOSS-I group was 70.3 ± 4.3 years, indicating no significant difference (**a**). The BMI of the FOSS-M group was 21.9 ± 0.3, which was significantly higher than that of the FOSS-I group, at 18.6 ± 1.2 (**b**)

This study consisted of 120 patients, 114 in the FOSS-M group and six in the FOSS-I group. The patients’ underlying conditions are shown in Table 1, and their T stages are shown in Table 2. The age and BMI of the two groups are shown in Fig. 1. Surgery was successfully completed in all patients. The characteristics of the patients in the FOSS-I group are summarized in Table 3. The mean age of the FOSS-M group was 70.3 ± 4.3 years and that of the FOSS-I group was 66.9 ± 1.0 years, indicating no significant difference. The BMI of the FOSS-M group was 21.9 ± 0.3 kg/m^2^, which was significantly higher than that of the FOSS-I group, at 18.6 ± 1.2 kg/m^2^.

**Table 2:** T stages of patients who underwent transoral surgery

Tstage	
Tis	30
T1	33
T2	23
T3	2
other	32

**Table 3:** Characteristics of patients with impaired oral intake after transoral surgery

Age	Primary	Subsite	TNM	BMI	RT	UC	Pre-PAS	Pre- FOSS	Post- FOSS
61	HP	PW	T2N2cM0	15.1	−	–	6	0	2
63	OP	LW	T1N2bM0	19.5	−	–	6	1	4
85	OP	BT	T3N0M0	20.9	+	COPD	6	2	3
77	LC	SG	T1N0M0	23.1	-	COPD	6	0	1
77	HP	PS	TisN0M0	16.7	+	COPD, PT	4	0	5
59	OP	UW	T2N0M0	15.8	+	–	4	1	2

*BMI* Body mass index, *RT* History of radiotherapy, *UC* Underlying conditions, *pre PAS* preoperative Penetration-Aspiration Scale, *FOSS* Functional Outcome Swallowing Scale, *HP* Hypopharyngeal cancer, *OP* Oropharyngeal cancer, *LC* Laryngeal cancer, *PW* Posterior wall, *LW* Lateral wall, *BT* Base of tongue, *SG* Supraglottic, *PS* Piriform sinus, *UW* Upper wall

### Parameters evaluated

**Fig. 2:**
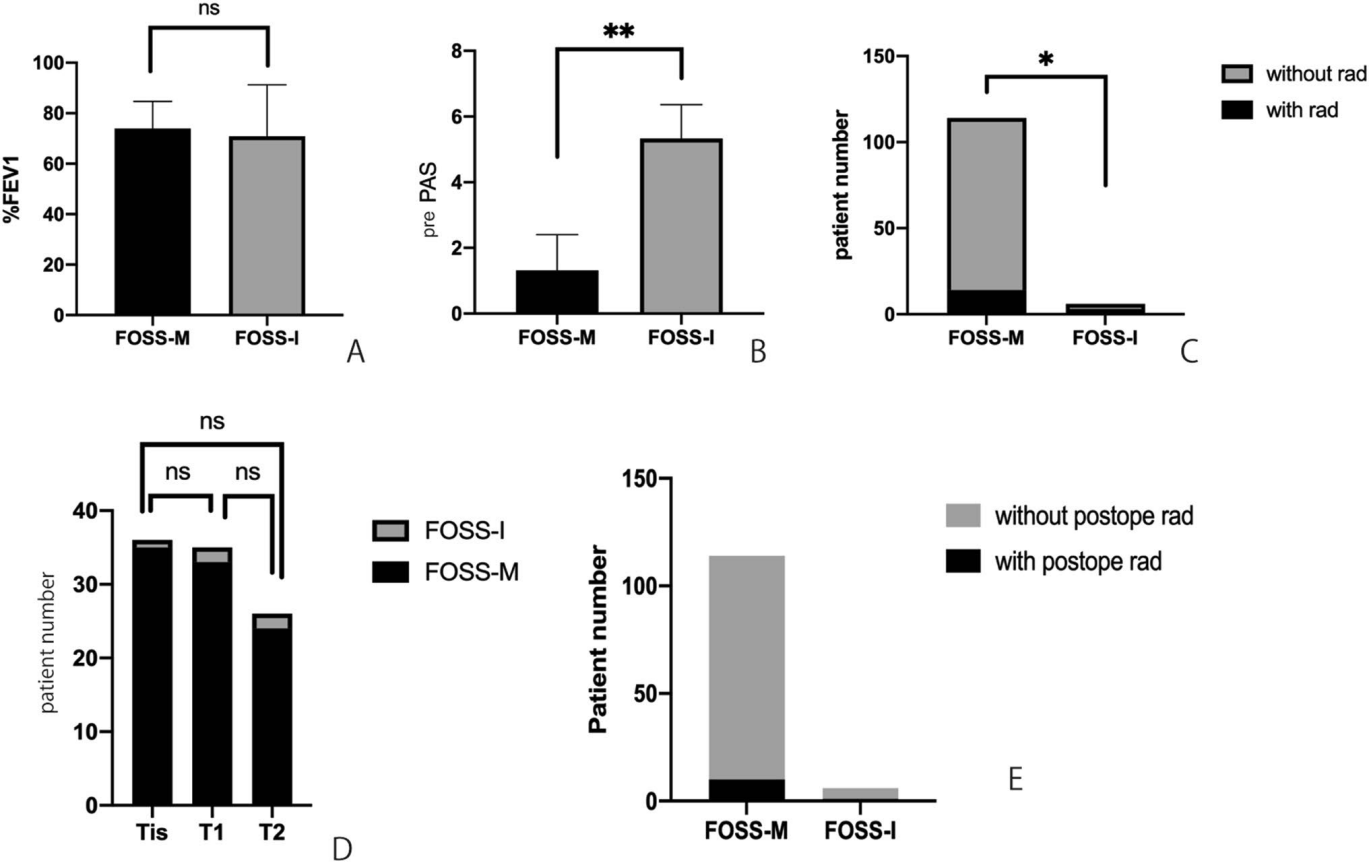
Comparison between groups regarding five perioperative factors: forced expiratory volume in 1 s (%FEV1), preoperative Penetration-Aspiration Scale (pre PAS), past history of radiotherapy for the head and neck, T stage, and postoperative radiotherapy. There was no significant difference in %FEV1 between the FOSS-M and FOSS-I groups (73.9 ± 1.0% vs 70.9 ± 8.3%, respectively) (**a**). On the other hand, pre PAS in the FOSS-M group was lower than that in the FOSS-I group (1.31 ± 0.1 vs 5.3 ± 0.4, respectively) (**b**). Fourteen of 114 (12.3%) patients in the FOSS-M group had a history of radiotherapy; this was a significantly lower rate than that in the FOSS-I group, in which three of six (50%) patients had a history of radiotherapy (**c**). In the FOSS-I group there was no significant difference in the percentages of patients with Tis, T1, and T2 (2.8% vs 5.7% vs 7.7%, respectively) (**d**). Postoperative radiotherapy was performed in five of 114 (4.4%) patients in the FOSS-M group, and in one of 11 patients (9.1%) in the FOSS-I group, indicating no significant difference (**e**)

Regarding preoperative spirometry, there was no significant difference in %FEV1 between the FOSS-M and FOSS-I groups (73.9 ± 1.0% vs 70.9 ± 8.3%, respectively) (Fig. 2a). On the other hand, preoperative PAS in the FOSS-M group was lower than that in the FOSS-I group (1.31 ± 0.1 vs 5.3 ± 0.4, respectively) (Fig. 2b). Fourteen of 114 (12.3%) patients in the FOSS-M group had a past history of radiotherapy, compared to three of six (50%) patients in the FOSS-I group, indicating a significant difference (Fig. 2c).

Regarding T stage, in the FOSS-I group, there was no significant difference in the percentages of patients with Tis, T1, and T2 (2.8% vs 5.7% vs 7.7%, respectively). Postoperative radiotherapy was performed in five of 114 (4.4%) patients in the FOSS-M group, and in one of 11 patients (9.1%) in the FOSS-I group, indicating no significant difference.

## Discussion

Compared to chemoradiotherapy and open partial laryngopharyngectomy, endoscopic transoral procedures provide excellent oncologic outcomes while preserving speech and swallowing function. Several studies reported satisfactory postoperative swallowing function, as indicated for example by a low rate of PEG dependency. However, in some cases, patients who undergo TOS experience persistent postoperative dysphagia. In this study, while swallowing function was similar before and after TOS in 114 patients, it worsened after surgery in six patients (5%). Few studies have focused on the risk factors for dysphagia following TOS. The present study is the first to use objective data, such as those derived from VF, to investigate this issue. VF is a standard examination performed at a number of hospitals, and is minimally invasive except for the risks posed by radiation exposure.

The current results showed that preoperative PAS was significantly correlated with impaired postoperative oral intake. Several studies reported that oral intake after surgery was associated with T stage and extent of resection [10]. Head and neck cancer was shown to be associated with an increased risk of malnutrition [11]. These patients might, therefore, have asymptomatic dysphagia that is initially identified during objective examinations. Surgery might make poor oral intake apparent. It is recommended that clinicians perform an objective examination preoperatively, for instance using VF.

In this study, a history of radiotherapy for head and neck cancer was significantly associated with impaired oral intake. Soft tissue fibrosis and sclerosis from radiotherapy influence laryngeal suspension. Inflammation and fibrosis that accompany irradiation can also alter muscle and nerve electrophysiology. In contrast to our findings, previous studies reported that a history of radiotherapy did not predict postoperative swallowing function [10, 12]. These studies assessed postoperative radiation in addition to a history of radiotherapy, and used the Eating Assessment Tool-10 for evaluation. In this study, adjuvant radiotherapy was also not associated with postoperative swallowing function. Regardless, it is advisable that clinicians take note of any history of radiotherapy.

In this study, BMI in patients with impaired oral intake was significantly lower than in those whose oral intake was preserved postoperatively. A previous prospective study reported that patients with weight loss of greater than 10% during the 6 months before surgery were at high risk of major postoperative complications [13]. Few studies have described the association between preoperative malnutrition and postoperative complications after TOS. This is the first study to report that preoperative malnutrition was associated with postoperative dysphagia after TOS.

Rich et al. reported that T4 advanced stage oropharyngeal cancer was associated with poor swallowing function after transoral laser microsurgery with or without adjuvant therapy [14]. Other studies found that arytenoid resection was associated with dysphagia [10, 15]. In the current study, T stage was not associated with poor swallowing function after TOS. However, this study included few patients with advanced T stage or invasion into the laryngeal cavity, and no patients who underwent arytenoid resection. Thus, this study could not demonstrate the relation between the extent and subsite of resection and postoperative swallowing function. This research focused only on preoperative factors. Further studies should evaluate the correlations of postoperative dysphagia with T stage, type of resection.

## Conclusion

This study suggests that patients with preoperative aspiration detected using VF might develop dysphagia severely after TOS. Other parameters, such as preoperative low BMI and a history of radiotherapy for head and neck cancer, were associated with postoperative dysphagia. Objective examination techniques such as VF should be performed preoperatively.
